# Co-Targeting Luminal B Breast Cancer with S-Adenosylmethionine and Immune Checkpoint Inhibitor Reduces Primary Tumor Growth and Progression, and Metastasis to Lungs and Bone

**DOI:** 10.3390/cancers15010048

**Published:** 2022-12-22

**Authors:** Ali Mehdi, Mikhael Attias, Ani Arakelian, Ciriaco A. Piccirillo, Moshe Szyf, Shafaat A. Rabbani

**Affiliations:** 1 Faculty of Medicine and Health Sciences, McGill University, Montreal, QC H3A 2B4, Canada; 2 Department of Human Genetics, McGill University, Montreal, QC H3A 2B4, Canada; 3 Program in Metabolic Disorders and Complications (MeDiC), Research Institute of the McGill University Health Centre, Montréal, QC H4A 3J1, Canada; 4 Department of Microbiology & Immunology, McGill University, Montreal, QC H3A 2B4, Canada; 5 Program in Infectious Diseases and Immunology in Global Health, Centre for Translational Biology, Research Institute of the McGill University Health Centre, Montréal, QC H4A 3J1, Canada; 6 Centre of Excellence in Translational Immunology (CETI), Montréal, QC H4A 3J1, Canada; 7 Department of Pharmacology and Therapeutics, McGill University, Montreal, QC H3A 2B4, Canada; 8 Department of Experimental Medicine, McGill University, Montreal, QC H3A 2B4, Canada; 9 Department of Oncology, McGill University, Montreal, QC H3A 2B4, Canada

**Keywords:** S-adenosylmethionine, luminal B, breast cancer, anti-PD-1 antibody, Eo771, bone metastasis

## Abstract

**Simple Summary:**

Breast cancer (BCa) is a devastating disease, which has a high prevalence and mortality in women. BCa metastasis is a major cause of mortality, and bone metastasis accounts for the majority of BCa-associated deaths. The luminal B subtype of BCa is immunogenically low and has the highest propensity to form bone metastasis compared to other BCa subtypes. Recent efforts have targeted BCa with immune checkpoint inhibitor (CPI) therapy. Although some clinical success in other BCa subtypes, luminal BCas had limited success. This has led to combining immune-stimulating therapies with CPIs to enhance the effectiveness of CPI therapy. We have demonstrated that a natural methyl donor, S-adenosylmethionine (SAM), has significant anti-cancer effects in various cancer models including all subtypes of BCa. Here, we show that SAM in combination with anti-PD-1 antibody has an enhanced anti-cancer efficacy compared to SAM, anti-PD-1 antibody, and control. The combination significantly reduced primary tumor growth and metastasis to lungs and bone. Hence, combining SAM with CPI has the potential to treat luminal B BCa.

**Abstract:**

Breast cancer (BCa) is the most prevalent cancer in females and has a high rate of mortality, especially due to increased metastasis to skeletal and non-skeletal sites. Despite the marked clinical accomplishment of immune checkpoint inhibitor (CPI) therapy in patients with several cancers, it has had limited success in luminal subtypes of BCa. Accordingly, recent efforts have focused on combination therapy with CPI, including epigenetic modulators, to increase response rates of CPI in luminal BCa. We have previously shown that S-adenosylmethionine (SAM), the ubiquitous methyl donor, has strong anti-cancer effects in various cancers, including all subtypes of BCa. In the current study, we took a novel approach and examined the effect of CPI alone and in combination with SAM on tumor growth and metastasis in a syngeneic mouse model of luminal B BCa. We showed that SAM decreases cell proliferation, colony-formation (survival), and invasion of luminal B BCa cell lines (Eo771, R221A) in vitro. In in vivo studies, in Eo771 tumor-bearing mice, either SAM or anti-PD-1 antibody treatment alone significantly reduced tumor growth and progression, while the SAM+anti-PD-1 combination treatment had the highest anti-cancer efficacy of all groups. The SAM+anti-PD-1 combination reduced the percentage of animals with lung metastasis, as well as total metastatic lesion area, compared to control. Additionally, the SAM+anti-PD-1 combination significantly reduced the skeletal lesion area and protected tibial integrity to a greater extent than the monotherapies in an Eo771 bone metastasis model. Transcriptome analysis of Eo771 primary tumors revealed significant downregulation of pro-metastatic genes, including Matrix metalloproteinases (MMPs) and related pathways. On the other hand, CD8^+^ T cell infiltration, CD8^+^ T cell cytotoxicity (elevated granzymes), and immunostimulatory genes and pathways were significantly upregulated by the combination treatment. The results presented point to a combination of SAM with CPI as a possible treatment for luminal B BCa that should be tested in clinical studies.

## 1. Introduction

Breast cancer (BCa) is now the primary cause of cancer incidence worldwide in females, with approximately 2.3 million new cases, or 11.7% of all cancers, in 2020 [1,2]. It has become the fifth leading cause of cancer-related death globally, accounting for more than half a million deaths annually [1,2]. High rates of morbidity and mortality are primarily due to increased metastasis to the lungs, brain, and especially to the skeleton [3]. The 10-year survival rate is reduced from 88% in stage I/II BCa to between 10 and 40% in stage III patients, with less than 10% for stage IV BCa [3]. In fact, metastasis accounts for approximately 90% of cancer-related deaths [4,5]. Therefore, there is an urgent need to reduce the morbidity and mortality, and the increase survival, of patients, especially those with metastatic BCa [3].

BCa is a heterogeneous cancer that has four major molecular subtypes: Luminal A (estrogen receptor (ER)^high^, progesterone receptor (PR)^high/low^, human epidermal growth factor receptor 2 (HER2)^low^); Luminal B (ER^high^, PR^high/low^, HER2^high/low^ki-67^high^); HER2 enriched (ER^low^, PR^low^, Her2^high^); Triple-negative BCa (ER^low^, PR^low^ and HER2^low^) [6]. Luminal A and B account for 70% of invasive BCa cases [2]. Compared to luminal A, luminal B tumors show higher proliferation and are associated with a higher cumulative incidence of distant metastasis [7,8]. Importantly, patients with the luminal B BCa subtype have the highest probability of forming bone metastasis, leading to skeletal-related events (SREs) that include hypercalcemia, intractable bone pain, nerve compression, and increased bone fragility, which collectively increase cancer-associated morbidity and mortality [9,10,11,12].

Immune checkpoint inhibitors (CPIs) increase CD8^+^ T cell anti-tumor activity and reverse the immune tolerant state [13,14,15]. Although the approval of CPI has led to a paradigm shift in cancer therapy, a significant proportion of patients do not respond and are resistant to CPI therapy, especially BCa patients [16,17]. The objective response rate to CPI is highly correlated to mutational burden (*p* < 0.001) [18,19]. BCa is generally considered to be less immunogenic due to a low mutational load (around 1/Mb) compared to melanoma and lung cancer (10/Mb) [19,20]. Moreover, BCa cases usually have high immunosuppressive regulatory T cell (Tregs) infiltration in the tumor microenvironment (TME) [21].

Luminal A and B are considered immunologically low; hence, the role of CPI therapy has been least investigated in these subtypes [22]. However, the luminal B BCa subtype may have certain immunological features that could increase sensitivity to CPI, including the expression of immune checkpoints, higher mutational load, and immune infiltration in TME, compared to luminal A [23]. In addition, the luminal B subtype was classified as an immune benefit subtype of BCa using breast tumor expression profiles and associated clinical data [24]. Furthermore, therapies including radiotherapy and chemotherapy that increase tumor mutational burden and immune infiltration into TME have the potential to enhance effectiveness of CPI against luminal B subtype BCa [22,23].

To distinguish CPI responders from non-responders, DNA hypomethylation has been identified as an essential biomarker that predicts low tumor response to host immunity and can also provide a mechanism for immune evasion and resistance to CPI [25]. These data are consistent with the idea that increased genomic methylation might restore CPI responsiveness. We have previously shown that S-adenosylmethionine (SAM), a ubiquitous methyl donor, targets DNA hypomethylation and blocks DNA demethylation, resulting in the downregulation of several essential oncogenes and pro-metastatic genes, including urokinase plasminogen activator (uPA) and matrix metalloproteinase-2 and 9 (MMP-2/9) [26,27]. SAM blocks BCa growth and metastasis in transgenic (MMTV-PyMT) and xenograft (MDA-MB-231) mouse models in a therapeutic setting [28,29,30,31]. Interestingly, SAM is also essential for T cell activation, proliferation, and survival [32,33,34,35,36,37,38,39,40]. SAM was also demonstrated to methylate the *FOXP3* gene, reducing *FOXP3* expression, hence, reducing the immunosuppressive capacity of Tregs [41]. In fact, a unique tumor immune escape mechanism that tumor cells use is depriving CD8^+^ T cells of methionine (a pre-cursor of SAM) in TME, which renders CD8^+^ T cells unable to produce cytokines and respond to CPI [42]. Lastly, SAM levels are reduced from the TME by tumor cells via various metabolic mechanisms [42,43].

Since cancer progression and metastasis involve a combination of multiple essential oncogenic and immune-suppressive pathways, CPI can be combined with other therapeutic agents, especially those targeting low immunogenic BCa subtypes [13,14,44]. Here, we used a novel therapeutic strategy by combining the methyl donor, SAM, with CPI and tested the anti-cancer efficacy on the luminal B BCa subtype that is immunogenically low and has a low response to CPI.

## 2. Results

### 2.1. SAM Decreases Proliferation, Colony Formation, and Invasion of BCa Cell Lines

**Figure 1:**
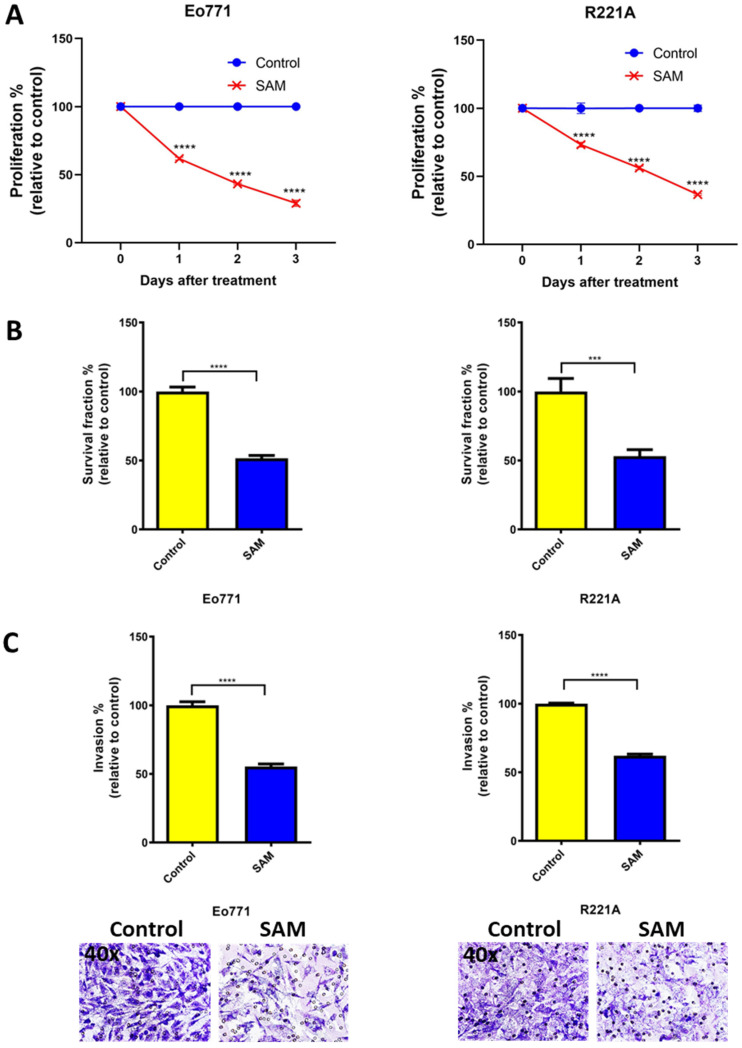
Effect of SAM on proliferation, colony-formation (survival), and invasion of luminal B BCa cell lines. (**A**) Percentage proliferation (± SEM) relative to control at 1, 2, and 3 days after SAM treatment. Briefly, Eo771 (4 × 10^4^) and R221A (1 × 10^4^) cells were seeded in 6-well plates, treatment with SAM (200 μM) started 2 days after seeding, and they were treated every day for 3 days. Cells were trypsinized and counted 1, 2, and 3 days after SAM treatment. (**B**) Percentage survival fraction (± SEM) relative to control obtained from soft agar colony formation assay. The colony formation assay was performed after the regular proliferation assay, and then the treated Eo771 (5 × 10^3^) and R221A (5 × 10^3^) cells were plated. Media was replenished every 4–5 days and colonies were counted after 3 weeks. (**C**) Invasion assay was performed after performing the regular proliferation assay and then incubating the treated cells (1.25 × 10^5^) for 18 h in two-compartment Boyden chambers coated with Matrigel. Top: Percentage invasion (± SEM) relative to control. Bottom: Representative images (lens, 40×; magnification, 400×) of invaded cells. Results are the mean of at least three independent experiments. Statistical significance was determined by (**A**) two-way ANOVA and (**B**, **C**) T-test in GraphPad prism. Significance values are represented by asterisks (*** *p* < 0.001; **** *p* < 0.0001).

One of the hallmarks of cancer is uncontrolled cellular proliferation [45]. Hence, first, we tested the anti-proliferative effects of SAM on two luminal B BCa cell lines, Eo771 and PyMT-R221A (R221A), using a well-established cell proliferation assay. SAM significantly decreased cell proliferation of both Eo771 and R221A cell lines compared to the untreated control (Figure 1A). The proliferation, relative to the control, was 62%, 43%, and 29% for Eo771 cells, and 73%, 56%, and 37% for R221A cells at day 1, 2, and 3 after the SAM treatment, respectively (Figure 1A). Next, we tested the effect of SAM on anchorage-independent growth of BCa cells using a soft agar colony formation (survival) assay. SAM significantly reduced the colony-forming potential of both Eo771 and R221A BCa cells, as the survival fraction for SAM-treated cells was 51% and 53%, relative to the control (100%), respectively (Figure 1B). Cancer metastasis processes require invasion and migration of the cancer cells through the extracellular matrix (ECM), and degradation and intravasation into the blood vessels. Thus, we tested the anti-metastatic effect of SAM using a robust cell invasion assay. Eo771 and R221A cells treated with SAM showed a significant reduction in cell invasive potential, as the invasion was 55% and 62% relative to the control (100%), respectively (Figure 1C). This data indicated that SAM reduces the proliferative, colony-forming (survival), and invasive ability of luminal B BCa cells.

### 2.2. Blocking Programmed Death Ligand 1 (PD-L1) Intrinsic Signalling Has No Effect on Cell Proliferation of BCa Cell Lines

**Figure 2:**
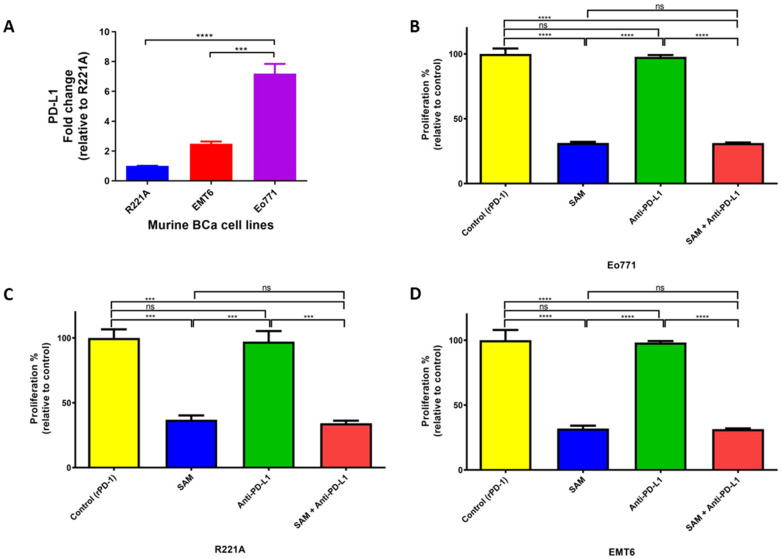
PD-L1 expression and effect of PD-L1 intracellular signaling on cell proliferation of murine BCa cells. (**A**) Expression of PD-L1 in murine BCa cell lines analyzed by RT-qPCR. The fold change was relative to the expression of R221A. (**B**–**D**) Effect of SAM and anti-PD-L1 antibody on proliferation of murine BCa cells. (**B**) Eo771 (4 × 10^4^), (**C**) R221A (1 × 10^4^), and (**D**) EMT6 (4 × 10^4^) cells were seeded in 6-well plates and were added to rPD-1 (0.2 μg/mL, day 3). The cells were treated with either control (only rPD-1), SAM (200 μM, day 2, 3, 4), anti-PD-L1 antibody (50 μg/mL, day 4), or SAM and anti-PD-L1 in combination. The results are the mean of at least three independent experiments. Proliferation is represented as the percentage proportional to the control (± SEM). Statistical significance was determined by one-way ANOVA in GraphPad prism. Significance values are represented by asterisks (ns; not significant; *** *p* < 0.001; **** *p* < 0.0001).

First, we established that PD-L1 is expressed in murine (Eo771, R221A, and EMT6) BCa cell lines, which are, therefore, candidates for CPI, wherein Eo771 cells showed the highest PD-L1 expression (Figure 2A). Although the major anti-cancer effects of blocking programmed cell death 1 (PD-1) and PD-L1 interactions in vivo are driven by the marked upregulation of anti-cancer adaptive immune responses, several studies have reported that PD-L1 also triggers intrinsic carcinogenic signaling independent of the immune checkpoint pathway [46,47]. We, therefore, tested the effects of SAM anti-PD-L1 antibody treatments and their combination on cellular proliferation. EMT6 is not a luminal B BCa cell line, but it has a moderate response to anti-PD-1/anti-PD-L1 antibodies in vivo and, thus, we tested for PD-L1 intrinsic signaling in EMT6 as well. The anti-PD-L1 antibody alone did not decrease cell proliferation in Eo771, R221A, and EMT6 cells (Figure 2B–D). The failure to decrease cell proliferation is consistent with previous reports indicating that the major effect of PD-1/PD-L1 blockade is through the rejuvenation of effector functions of CD8^+^ T cells and its tumor lytic activity [14,48]. Our results showed that PD-L1 intrinsic signaling does not affect cell proliferation. The decrease in cellular proliferation in the SAM+anti PD-L1 combination group was probably only due to SAM in the luminal B BCa cell lines.

### 2.3. The SAM and Anti-PD-1 Antibody Combination Has a Superior Effect in Reducing Primary Breast Tumor Growth Compared to Monotherapies

The luminal B subtype of BCa has the highest propensity to metastasize to the lungs and skeletal system amongst the BCa subtypes [9,10,11,12]. Eo771 BCa cells show a molecular pattern of ERα^−^, ERβ^+^, PR^+^, and ErbB2^+^, and have recently been characterized as the luminal B subtype [49,50]. Eo771 tumor-bearing syngeneic mice can be established by inoculating Eo771 cells in immunocompetent C57BL/6 mice. Furthermore, this model offers the advantage of studying lung metastasis naturally derived from primary tumors. We first established the tumor growth kinetics of Eo771 tumor-bearing mice by injecting varying numbers of Eo771 cells orthotopically into female C57BL/6 mice at the 4th mammary fat pad (m.f.p). We found that the tumors started to ulcerate from day 22–24 post-inoculation, and we, therefore, set the timeline of our experiments for 20 days post-inoculation.

**Figure 3:**
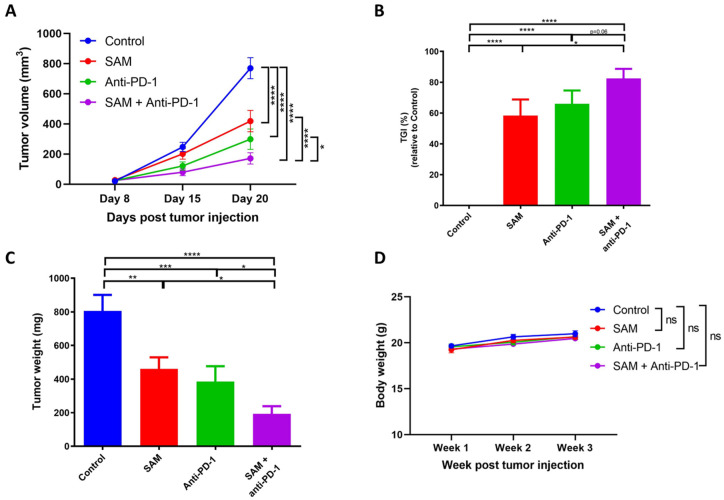
SAM, anti-PD-1 antibody, and the combination treatment decreased primary tumor growth in Eo771 tumor-bearing mice. (**A**) Eo771 (2 × 10^5^ cells) were injected at the 4th m.f.p in B6 mice to induce tumor formation. The animals were treated with either the control (isotype matched IgG and PBS), SAM (80 mg/kg/day), anti-PD-1 antibody (5 mg/kg, twice per week), or combination. Tumor volumes were assessed at day 8, 15, and 20, and the animals were sacrificed at day 20. Results are presented as the mean ± SEM of tumor volume (*n* ≥ 7/group). (**B**) Percentage tumor growth inhibition (TGI) was calculated from tumor volumes at day 15 to day 20, relative to the control. (**C**) Tumor weight (mg ± SEM) was measured after tumor harvest on day 20. (**D**) Body weight (g ± SEM) of the mice was measured once a week. Statistical significance was determined by (**A**, **D**) two-way ANOVA; (**B**, **C**) one-way ANOVA in GraphPad prism. Significance values are represented by asterisks (ns, not significant; * p < 0.05; ** *p* < 0.01; *** *p* < 0.001 and **** *p* < 0.0001).

Treatment with either SAM or anti-PD-1 antibody significantly reduced tumor growth and progression in Eo771 tumor-bearing animals compared to the control at day 20 post-tumor injection. However, the SAM+anti-PD-1 antibody combination had the highest efficacy in controlling tumor growth and progression at day 20 post-tumor injection compared to all groups, as indicated by the least mean tumor volume and tumor weights (Figure 3A,C). The combination treatment also had the maximum percentage of tumor growth inhibition (TGI) at 82% compared to the control (0%), SAM (58%), and anti-PD-1 antibody (66%), although the TGI difference between the combination treatment and anti-PD-1 antibody alone was barely significant (Figure 3B). We found no difference in the mouse body weights between the control and treatment groups (Figure 3D).

### 2.4. The SAM and Anti-PD-1 Antibody Combination Decreases Lung Metastasis

**Figure 4:**
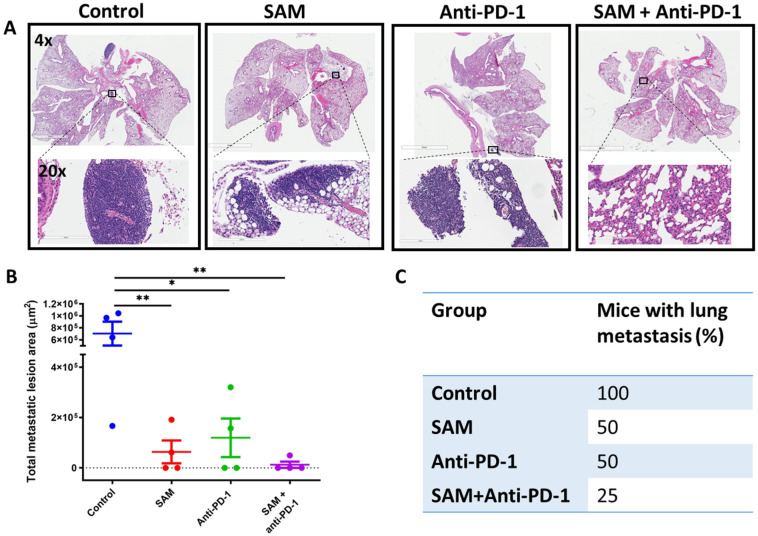
SAM, anti-PD-1 antibody, and the combination treatment decreased lung metastasis in Eo771 tumor-bearing mice. Briefly, mice were injected with Eo771 cells orthotopically at the m.f.p and treated with the four treatments indicated. At the end of the study, lungs of the mice were harvested, fixed using formalin, embedded in paraffin, sliced, and stained with H&E. (**A**) Representative histology images of mouse lung showing the whole lung and magnified images to show metastatic lesions from each group except the SAM+anti-PD-1 antibody combination group, which had no lesions in this sample. Lens: top; 4×; bottom; 20×. Magnification: top; 40×; bottom; 200×. (**B**) Total metastatic lesion area (µm^2^ ± SEM) for each group (*n* = 4/group). Total metastatic lesion area was calculated by annotating all the metastatic lesions in the entire lung of a mouse using the ImageScope annotation tool, which gives the selected area. Then, all the lesion areas were added together. (**C**) Percentage of mice with lung metastasis in each group. Statistical significance was determined by (B) one-way ANOVA in GraphPad prism. Significance values are represented by asterisks (* p < 0.05; ** *p* < 0.01).

Lung metastasis is a common feature of luminal B BCa [9,10,11,12]. Accordingly, we investigated the effect of SAM and anti-PD-1 antibody on lung metastasis using the Eo771 BCa mouse model. The Eo771 primary tumor cells followed the progressive steps of metastasis and formed metastatic lesions in the lungs. At the end of these studies, on day 20 post-Eo771 tumor cells inoculation, the animals were euthanized, and the lungs were harvested, fixed, permeabilized, and stained with Hematoxylin and Eosin (H&E). Lungs of animals treated with SAM and anti-PD-1 antibody alone showed a significant decrease in the total metastatic lesion area compared to the lungs of the control animals (Figure 4A,B). Furthermore, lungs of animals that were treated with the SAM and anti-PD-1 combination showed the smallest total metastatic lesion area compared to all groups, though the difference did not reach statistical significance compared to either monotherapy (Figure 4A,B). Additionally, the combination treatment had the lowest percentage (1/4 mice; 25%) of mice with lung metastasis compared to SAM (2/4 mice; 50%), anti-PD-1 antibody (2/4 mice; 50%), and control (4/4 mice; 100%) (Figure 4C).

### 2.5. The SAM and Anti-PD-1 Antibody Combination Blocks Bone Metastasis and Protects Bone from Tumor Osteolytic Damage

Bone metastasis is a common feature of BCa, wherein tumor osteolytic lesions are formed, which leads to SREs and a poorer prognosis for BCa patients. Moreover, luminal B BCa has the highest propensity to metastasize to skeletal sites [9,10,11,12]. Therefore, we evaluated the effect of the SAM+anti-PD-1 combination on skeletal metastasis. We first established a syngeneic luminal B bone metastasis model by injecting several doses of Eo771 cells into the tibia of black B6 mice intra-tibially (i.t). Ten thousand Eo771 cells were determined to be optimal for forming bone lesions and provided a sufficient therapeutic window of 21 days.

**Figure 5:**
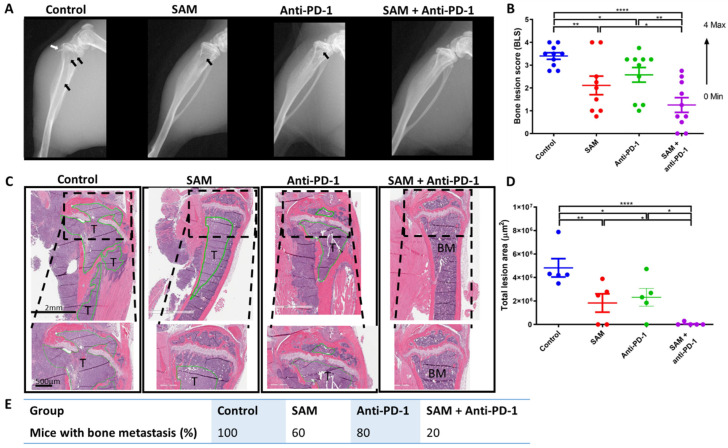
The SAM and anti-PD-1 antibody combination decreases bone metastasis and protects the bone from damage caused by aggressively growing tumor lesions. Briefly, mice were injected with Eo771 cells intra-tibially and treated with either control (isotype matched IgG and PBS, *n* = 10/group), SAM (80 mg/kg/day, *n* = 9/group), anti-PD-1 antibody (5 mg/kg, twice per week, *n* = 10/group), or the combination (*n* = 10/group). (**A**) Representative X-ray images showing the anatomy of the lower limb. The tibia, fibula, and femur (in part) along with the knee joint are shown. X-rays of the mice were taken at day 21 post-tumor injection. Black arrows indicate tumors, while white arrows indicate a broken cortical bone margin. (**B**) X-ray images were used to calculate a bone lesion score (BLS) for each group in increments from 0 to 4, where 0 represents no tumor lesions with the highest bone integrity (no breaks in the peripheral margin) and 4 represents the maximum tumor lesion area with the lowest bone integrity and with major breaks in the peripheral margin (*n* = 10/group, except SAM (*n* = 9/group)). (**C**) Representative histology images of mouse tibias 21 days post-tumor injection. Briefly, mice were sacrificed at day 21, and tibias were extracted, fixed, decalcified, embedded, sliced, and subjected to H&E staining, as described in Materials and Methods. T, tumor; BM, bone marrow. The black bar at the bottom left represents the scale in each image: top, 2 mm; below, 500 µm. (**D**) Total bone lesion area (µm^2^) for each group (*n* = 5/group). Briefly, the tumor lesion area in the whole tibia image was measured using the ImageScope annotation tool, added and plotted in GraphPad Prism. (**E**) Percentage of mice with bone metastasis in each group. Statistical significance was determined by (**B**, **D**) one-way ANOVA in GraphPad prism. Significance values are represented by asterisks (* p < 0.05; ** *p* < 0.01; **** *p* < 0.0001).

After establishing the bone metastasis model, we investigated the impact of SAM, anti-PD-1 antibody, and the combination on metastasis of Eo771 BCa cells within the bone microenvironment and treated these animals for 21 days. Skeletal lesions were calculated using a bone lesion score (BLS) as described in the Section 4. Digital radiography showed the highest BLS for the control animals, followed by significantly lower BLS for anti-PD-1 antibody and SAM alone (Figure 5A,B). Moreover, the tibias of animals treated with the SAM+anti-PD-1 combination scored significantly lower for BLS as compared to the other groups (Figure 5A,B).

To confirm the X-ray results, we harvested the tibias of the animals, fixed and decalcified the bones, and carried out H&E staining. The tumor lesions in the tibias of the control group digested the cortical bone outside the tibia, as well as through the growth plate into the epiphysis upwards, and metaphysis and diaphysis downwards (Figure 5C). For all control group tibias, most of the cortical bone was broken and the tumor had grown into the whole bone marrow. To be consistent, we measured the tumor lesion area only within the tibia. SAM and anti-PD-1 antibody alone had a significant effect on controlling tumor lesion growth within the tibia compared to the control, as the tumor lesions were smaller (Figure 5C,D). However, the combination of SAM and anti-PD-1 antibody significantly reduced tumor lesion growth compared to monotherapies, as indicated by the smallest lesion area compared to all groups (Figure 5C,D). The combination treatment also protected the bone as the cortical bones were thicker, and the tissues, including bone marrow, epiphysis, growth plate, metaphysis, and diaphysis, were intact, mirroring the histology of a normal mouse tibia (Figure 5C). Lastly, the combination treatment had the lowest percentage (20%) of mice with bone metastasis compared to SAM (60%), anti-PD-1 (80%), and control (100%) (Figure 5E).

### 2.6. The SAM and Anti-PD-1 Antibody Combination Reduces Expression of Oncogenes While Elevating Expression of Immunostimulatory Genes, as well as CD8^+^ T Cell Infiltration and Activity

**Figure 6:**
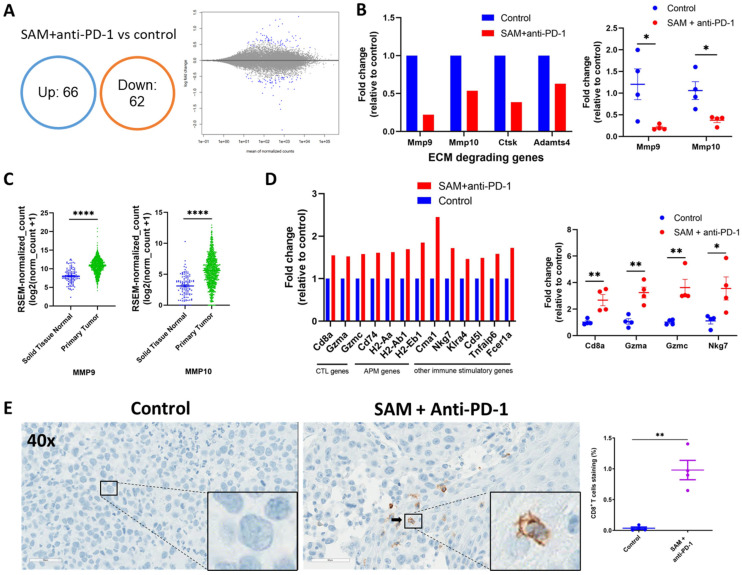
Tumors treated with the SAM and anti-PD-1 antibody combination show reduced expression of key oncogenes and pro-metastasis genes, and elevated expression of immunostimulatory genes. (**A**) Venn diagram (left) and MA plot (right) showing significant DEGs (*p* < 0.001) in SAM and anti-PD-1 antibody combination-treated Eo771 tumors versus control Eo771 tumors. Up, upregulated genes; down, downregulated genes. (**B**) Change in expression of significantly downregulated genes in the combination-treated versus control tumors extracted from RNA-seq data (left, *n* = 3/group) and validated with RT-qPCR (right, *n* = 4/group). The data are presented as fold change in expression in the treatment group relative to the control. The value of the control was set at 1. (**C**) Expression of key pro-metastatic genes *MMP9* and *MMP10* in human solid normal tissue and primary tumor tissue of breast cancer patients derived from GTEx and TCGA databases (*n* = 1391 samples) using the Xena platform. Expression values are depicted in RSEM, which is RNA-Seq by Expectation Maximization. (**D**) Change in expression of top significantly upregulated genes in combination-treated versus control tumors extracted from RNA-seq data (left, *n* = 3/group) and validated with RT-qPCR (right, *n* = 4/group). Data is presented as fold change in the treatment group relative to the control. The value of the control was set at 1. CTL, cytotoxic T lymphocytes; APM, antigen processing and presentation machinery. (**E**) Immunohistochemistry with CD8a^+^ T cell marker staining of Eo771 tumors treated with the combination treatment and the controls. (**E**, left) Representative images (lens, 40×; magnification, 400×) of the primary Eo771 tumors stained with murine antibody against CD8a^+^ marker (brown) from the control and SAM+anti-PD-1 antibody combination-treated tumors. The nuclei are stained blue and a CD8^+^ T cell is indicated by a black arrow. Enlarged images at the bottom right show the absence and presence of CD8^+^ T cells in the control and SAM+anti-PD-1 antibody group, respectively. (**E**, right) CD8a^+^ T cell positive staining area percentage (*n* = 4 samples/group). Statistical significance was determined using (**C**,**E**) T-test in GraphPad prism and (**A**,**B**,**D**) by Wald test with BH FDR (*≤ 0.2*) correction. Significance values are represented by asterisks (* p < 0.05; ** *p* < 0.01; **** *p* < 0.0001).

To determine the reason for the significant reduction in tumor growth and progression and metastasis due to the combination treatment, we carried out RNA-sequencing analysis of the Eo771 tumors treated with the combination treatment and the control. RNA-seq revealed that 128 protein coding genes were differentially expressed in the combination group as compared to the control (Figure 6A, Supplementary File S1).

Matrix metalloproteinases (MMPs) are endopeptidases that are involved in tumor growth, progression, and metastasis [51,52]. The significantly top downregulated genes were enriched in pathways related to the organization of extracellular matrix (ECM), activation of MMPs, and degradation of the ECM (Figure 6B and Supplementary Figure S1). Specifically, *Mmp9*, *Mmp10, Adamts4,* and *Ctsk,* which are involved in degradation of the ECM, were downregulated in the combination treatment group (Figure 6B). We also validated the downregulation of *Mmp9* and *Mmp10* using RT-qPCR (Figure 6B). We carried out clinical database analysis of the TCGA and GTEx databases using the Xena platform [53]. We found that both *Mmp9* and *Mmp10* genes are upregulated in primary tumors of human breast cancer patients compared to normal solid tissue (Figure 6C).

CD8^+^ T cells control tumor growth by killing tumor cells directly using proteases, including granzymes, upon activation, and becoming cytotoxic effector cells [54,55,56]. We found that the top significantly upregulated genes were involved in antigen processing and presentation, immunostimulatory molecules, granzymes, and genes that provide a high response to CPI therapy (Figure 6D). Firstly, the number of CD8^+^ T cells was increased in the TME of combination-treated tumors, as indicated by increased *Cd8a* expression. Secondly, different genes (including *Cd74*, *H2-Aa*, *H2-Ab1*, *H2-Eb1*) of antigen processing and presentation machinery (APM) were increased in combination-treated tumors. Thirdly, the granzymes (*Gzma* and *Gzmc*) that are released by CD8^+^ and NK cells to kill tumor cells had a higher expression in combination-treated tumors compared to control. Fourthly, a key immunostimulatory gene, *Nkg7*, whose elevation has been associated with good response to CPI therapy in multiple studies [57,58], was also upregulated in the combination treatment group. Moreover, other immune-stimulating genes, including *Cma1,* were upregulated as well. We also validated the upregulation of key genes, including *Cd8a, Gzma*, *Gzmc,* and *Nkg7,* using RT-qPCR (Figure 6D). Lastly, we found that the top significantly upregulated pathways were involved in elevating anti-cancer immune responses, such as antigen processing and presentation, Th1, Th2, and Th17 differentiation, Allograft rejection, and other immune stimulating pathways (Supplementary Figure S2).

We also carried out immunohistochemistry (IHC) of the Eo771 tumors from groups treated with the combination and controls. We performed H&E staining and staining for the CD8^+^ T cell marker in order to determine the extent of CD8^+^ T cell infiltration in either the Eo771 tumors treated with the combination or the control group. We found a higher number of CD8^+^ T cells in the tumors that were treated with the combination treatment compared to the control (Figure 6E). The higher CD8^+^ T cell infiltration together with higher granzymes expression data could indicate higher activation and function of CD8^+^ T cells.

Taken together, these data indicate that treatment with the SAM and anti-PD-1 antibody combination reduces tumor growth and progression by downregulating oncogenes, elevating immune responses, and upregulating immunostimulatory genes.

## 3. Discussion

Although CPIs are considered a breakthrough in cancer treatment, they have limited therapeutic effect in less immunogenic cancer types such as luminal B BCa [22,23]. Furthermore, CPI therapy causes adverse effects. Hence, current therapies have evolved to combine CPI therapy with other agents to enhance the CPI response and reduce the toxicity of CPI. In the current study, we examined a new approach that involves combining a methyl donor, SAM, with CPI to enhance responses to immunotherapy in the luminal B BCa subtype. The luminal B subtype was studied here since it has the lowest response rates to CPI and highest propensity to form bone metastasis compared to other subtypes of BCa [9,10,22,23]. In in vitro studies, SAM was effective in inhibiting several cancer growth and invasion parameters in luminal B BCa cell lines. The reduction in proliferation by SAM was reported to be due to the downregulation of cyclins, upregulation of cell cycle inhibitors, and/or downregulation of Jak/Stat pathways in prostate cancer, osteosarcoma, gall bladder carcinoma, and pancreatic cancer [59,60,61,62,63,64]. We believe the mechanism of repressed proliferation by SAM could be similar in luminal B BCa. In in vivo studies, either SAM or anti-PD-1 antibody on their own reduced primary tumor growth of a syngeneic Eo771-tumor bearing mouse model compared to control mice. However, the combination treatment had the highest reduction in tumor growth and progression, and reduced metastasis to the lungs and bones.

We chose the Eo771 model for our in vivo studies for the following reasons. Firstly, Eo771 cells have the highest expression of PD-L1 amongst the luminal B cell lines (Figure 2A) [49,50]. Secondly, we found that Eo771-tumor bearing mice show immune infiltration in the TME. Thirdly, the Eo771 cell line is one of the few syngeneic luminal B BCa subtypes that can form primary tumors as well as metastasize to skeletal and non-skeletal sites with characteristics similar to the human disease [65]. Fourthly, Eo771 cell inoculation has a consistent (100%) tumor uptake/penetrance [66]. However, Eo771 tumor-bearing mice form ulcers at from around 22–25 days following tumor inoculation at the m.f.p, therefore, the animals were sacrificed on day 20 in accordance with McGill University guidelines. Due to the short experimental therapeutic window of this model, we could not assess the long-term effects of SAM and anti-PD-1 antibody. Nevertheless, based on the established efficacy of SAM and anti-PD-1 antibody in several cancers, including melanoma, we anticipate continued benefit and effectiveness of SAM+anti-PD-1 in a luminal B BCa model, which could be translated to a clinical setting [28,30].

In patients with luminal B BCa, skeletal metastasis is a major complication that ultimately leads to SREs [9,10,11,12]. However, limited therapeutic options are available and most of the therapies are palliative, targeting bone pain reduction and reversing bone resorption [67]. We, therefore, examined whether the combination therapy might address this challenge. We first established a bone metastasis model by injecting Eo771 cells via the intra-tibial route [68,69]. Then, we evaluated the effect of SAM, anti-PD-1 antibody, and the combination of both on the formation of bone metastatic lesions. We found that SAM and anti-PD-1 antibody alone could significantly reduce the ability of Eo771 cells to form metastatic lesions. Importantly, SAM in combination with anti-PD-1 antibody had the highest effect in reducing tumor cells in the tibia and resulted in a reduced lesion area. Following histological analysis, animals treated with the combination therapy showed higher integrity and the tibia was similar to a normal mouse tibia, suggesting rapid clearance of tumor cells from the bone microenvironment by the combination treatment.

MMPs are endopeptidases that function as proteolytic enzymes. High expression of MMPs in tumors allow them to degrade ECM proteins and the basement membrane to invade and metastasize to nearby tissues and distant organs [51,52]. Apart from being key players in the metastasis of cancer cells, MMPs are also linked to tumorigenesis due to their functions in proliferation, apoptosis, and angiogenesis [51,52]. A meta-analysis comprised of 41 studies and 6517 breast cancer patients demonstrated that a higher expression of *Mmp2* and *Mmp9* in tumor cells of patients was strongly associated with larger tumors and metastasis to lymph nodes and distant organs [52]. Higher expression of *Mmp2* and *Mmp9* was also associated with histological grade, higher clinical stage, and predicted poor survival of breast cancer patients [52]. The SAM and anti-PD-1 antibody combination-treated tumors had significant downregulation of the *Mmp9* gene (and other MMP-related genes) and pathways involved in ECM degradation and activation of MMPs. Our lab also demonstrated that SAM reduces the expression of pro-metastatic genes, including MMPs in BCa, prostate cancer, and osteosarcoma [27,31,62,70]. The results reported herein and previously published together are consistent with the idea that the reduction in tumor growth and progression, and metastasis to skeletal and non-skeletal sites, could be attributed to the effects on these well-established oncogenic and metastatic pathways.

CD8^+^ T cell activation depends upon engagement of the CD8 receptor with antigens presented by MHC class I. MHC I and II complexes are mutated or downregulated in several cancers, and this is a major tumor immune evasion mechanism used by tumor cells to avoid immune destruction [71]. Therefore, elevated antigen processing and presentation (by MHC class I/II), increased immunostimulatory molecules, and increased production of granzymes are major anti-cancer immune mechanisms and factors that determine the success of CPIs [72]. Upregulation of anti-cancer immunity, especially CD8^+^ T cell infiltration, activation, and effector functions by the SAM and anti-PD-1 antibody combination, has been reported by us in a melanoma mouse model [48]. This observation was also true in the current study, where CD8^+^ T cell infiltration and effector functions, such as higher granzyme production, were elevated upon SAM and anti-PD-1 antibody combination treatment. Parallel to this, methionine (pre-cursor of SAM) supplementation restored CD8^+^ T cell immunity in melanoma and ovarian murine tumors [42]. However, the enhancing effect of SAM on anti-cancer immunity was not observed in hepatocellular carcinoma, where SAM led to T cell exhaustion [73]. The effect of SAM on anti-cancer immunity could be cancer-type- or TME-dependent. To address this question, future studies could examine the effect of SAM alone and with anti-PD-1 antibody in combination on CD8^+^ T cells and other immune cells in TME through single-cell RNA sequencing analysis.

Although SAM has been delivered via different routes in other studies, we found that delivery of SAM via oral gavage has several advantages. Firstly, oral gavage provides the ability to accurately control the actual amount of SAM given to each mouse, as compared to adding SAM into drinking water, in which case the precise amount for each mouse cannot be controlled. Secondly, in previous studies, we found that the oral route resulted in elevated levels of SAM in the mice serum [28,30]. Thirdly, this method avoids eliciting an immune response and effectively avoids injection of SAM into different tissues/organs, a potential risk associated with i.p. injections. Lastly, SAM is non-toxic, is already an approved nutraceutical agent, and treatment with SAM has not been shown to trigger adverse effects in pre-clinical and clinical studies [28,30,31,48,74]. Therefore, SAM can be given in a combination setting and continued thereafter in a non-hospital setting following the successful completion of CPI in BCa patients to block tumor progression, metastasis, and BCa-associated morbidity, which remain our ultimate goal.

## 4. Materials and Methods

### 4.1. Cell Lines

Murine BCa Eo771 and PyMT-R221A (R221A) cell lines were generously provided by Dr. Conor C. Lynch (H. Lee Moffitt Cancer Center and Research Institute, Tampa, FL, USA) and Dr. Jean S. Marshall (Dalhousie University, Halifax, Nova Scotia, Canada), respectively. The EMT6 mouse BCa cell line was obtained from ATCC (Manassas, Virginia). Eo771. R221A and EMT6 cells were cultured in DMEM supplemented with 10% FBS, 1% penicillin-streptomycin sulfate, HEPES, and 2 mM L-glutamine. All the cell lines used were of early passage, were found to be mycoplasma free, and were maintained in incubators at 37 °C and 5% CO_2_.

### 4.2. Proliferation, Soft Agar Colony Formation, and Invasion Assays

For in vitro studies, we used 200 μM of SAM (cat# B9003S, NEB, Mississauga, Canada) and 50 μg/mL of anti-PD-L1 antibody (clone 10F.9G2, cat# BE0101, BioXcell, Lebanon, NH, USA), as these concentrations were found to be optimal in our previous dose response studies [26,28,48,62,63,75,76].

For the proliferation assay, Eo771 (4 × 10^4^), EMT6 (4 × 10^4^) and R221A (2.5 × 10^4^) were seeded in 6-well plates. Cells were treated with SAM (200 μM) on day 2, 3, and 4 and were collected on day 5. On day 5, the treated cells were trypsinized, mixed with complete DMEM, and counted using Beckman Coulter (Hertfordshire, UK). Percentage proliferation (%) was calculated as (mean number of cells in treatment group/mean number of cells in control group) × 100. To determine the effect of intracellular signaling of PD-L1 on cellular proliferation, the Eo771 (4 × 10^4^), EMT6 (4 × 10^4^), and R221A (2.5 × 10^4^) cells were seeded and treated with recombinant PD-1 (rPD-1, Control), SAM, anti-PD-L1 antibody, and SAM+anti-PD-L1. Eo771, EMT6, and R221A cells were supplemented with 0.2 μM rPD-1 (cat# 1021-PD-100, R&D systems, Minneapolis, MN, USA) on day 3, which was followed with 50 μg anti-PD-L1 on day 4. The treated cells were counted on day 5.

Soft agar colony formation assay followed the regular proliferation protocol for SAM treatment and was carried out as previously described [28,30]. Briefly, Eo771 (5 × 10^3^) and R221A (5 × 10^3^) SAM- and control-treated cells in complete DMEM media supplemented with 13% FBS were mixed with 0.6% agar. This cell–agar mixture was seeded and allowed to solidify on top of another 2% agar solidified layer in a 6-well plate. Media was added on top and replenished every 4–5 days. After 3 weeks, the colonies were counted under a light microscope. A group of at least 50 cells that were not overlapping was considered a colony and the percentage survival fraction calculated as previously described [28,30]. Invasion assay followed the regular proliferation protocol for SAM treatment and was run precisely as detailed by us previously, using a two-compartment Boyden chamber coated with Matrigel (Sigma-Aldrich, Oakville, ON, Canada) [28,48]. The Eo771 and R221A cells were allowed to invade for 18 h. Percentage invasion (%) was calculated as (mean number of cells invading per field in SAM or control group/mean number of cells invading per field in control group) × 100. All assays are presented as the average of at least three independent repeats.

### 4.3. Animal Studies

Female C57BL/6 mice (six to eight weeks old) were obtained from Charles River Lab (Quebec, Canada) and kept at an ARD facility of the RI-MUHC. Mice were injected orthotopically with 2 × 10^5^ Eo771 cells at m.f.p to form tumors. The animals were randomized into the four treatment groups, including control (isotype matched IgG2a and PBS), SAM (Life Science Laboratories, Lakewood, NJ, USA), anti-PD-1 antibody (clone RMP1-14, BioXcell, Lebanon, NH, USA), and SAM+anti-PD-1 combination. SAM (80 mg/kg) was diluted in PBS (1×) and was given daily using feeding needles via oral gavage once the tumors became palpable, and anti-PD-1 antibody (5 mg/kg) and isotype matched IgG2a antibody (5 mg/kg) were administered via intra-peritoneal (i.p.) injection twice a week. The dose of SAM and anti-PD-1 antibody was established previously [13,28,48,77,78,79]. Tumor volume (T.V) was measured by palpation at timed intervals using a digital caliper. On day 20, the mice were sacrificed and tumor weight and T.V were measured. T.V was calculated using the formula T.V = (length × width^2^)/2. Percentage (%) tumor growth inhibition was calculated as ((1–[changes of T.V in treatment group/changes of T.V in control group] × 100). The mice were observed regularly for weight loss or potential adverse effects [80].

### 4.4. RNA Extraction and Reverse Transcriptase Quantitative Real-Time PCR (RT-qPCR)

Total RNA was isolated using the RNeasy kit (cat# 71404, Qiagen, Hilden, Germany). The RT-qPCR assay was run as previously described by us [28,48]. Gene expression changes were analyzed by following the 2-ΔΔCT method. The primer list has been tabulated in Supplementary Table S1.

### 4.5. RNA-Sequencing (RNA-Seq) and Analysis

The total RNA was extracted from Eo771 breast tumors and subjected to quality control (QC) using Bioanalyzer and NanoDrop. An A260/280 absorbance ratio of >2.0 and RIN of >6.5 qualified the samples for RNA-seq. After QC, paired-end RNA-seq was carried out using the Illumina NovaSeq 6000 platform (with a depth of 25 million reads) following the standard methodology by Genome Quebec (McGill University). The raw sequencing data was checked for QC, normalized, and used to generate HT-seq count files. The HT-seq files were then input into the DeSeq2 tool (RRID:SCR_015687) to carry out the differential gene expression (DEGs) analysis (*FDR ≤ 0.2*) in Galaxy (www.usegalaxy.org). Pathway enrichment analysis of DEGs was performed by ConsensusPathDB (RRID:SCR_002231).

### 4.6. Intratibial Model for Skeletal Metastasis

Firstly, a murine luminal B BCa bone metastasis model was established by implanting varying doses of Eo771 cells (2 × 10^5^, 1 × 10^5^, 0.5 × 10^5^, 0.2 × 10^5^,0.1 × 10^5^) into the tibia of female C57BL/6 mice (six to eight weeks old) using 27G surgical needles. Briefly, the needles were inserted intra-tibially into the tibial region of the mice via the knee joint in a clockwise rotation. Once the needles were completely inserted into the bone, the cells were slowly released into the bone microenvironment. After detailed assessment, we found that 0.1 × 10^5^ Eo771 cells were optimal for our experiments, as this provided a decent therapeutic window; this concentration was bearable for the mice as the bones were not fragile upon harvest and the knee joints were intact upon harvest for most mice. Then, we assessed whether SAM, anti-PD-1 antibody, and their combination can reduce tumor cell growth within the bone microenvironment of these models. Briefly, Eo771 (0.1 × 10^5^) cells were injected via intra-tibial route and the mice were randomized two days post-tumor implantation. The mice were treated with either vehicle (IgG2a via i.p. injection twice a week and PBS), SAM (via oral gavage daily), anti-PD-1 antibody (via i.p. injection two times a week), or the combination until sacrifice on day 21 (*n*  =  10/group). Digital radiography of hind limbs was performed at day 21 using Bruker In-Vivo Xtreme, according to standard protocol at the RI-MUHC. Skeletal lesions were analyzed and given a BLS as previously described by us and others [81,82,83]. The BLS was averaged from two separate histologist scorings. Briefly, the BLS was determined from 0–4 as follows: 0 represents no tumor lesions with highest bone integrity (no breaks in the peripheral margin); 1 represents minor lesions; 2 represents small lesions; 3 represents considerable tumor lesions with minor breaks in peripheral margin; 4 represents the maximum tumor lesion area with lowest bone integrity and with major breaks in the peripheral margin [81,82,83].

### 4.7. Immunohistochemistry (IHC)

The lungs of animals treated with the control (IgG2a and PBS), SAM, anti-PD-1 antibody, and the combination were harvested at the endpoint. The lungs were fixed with formalin for between 3 and 5 days, washed with 70% ethanol, embedded in paraffin, sectioned, and stained with H&E staining. Following the intratibial model of bone metastasis, tibias were harvested at the endpoint (day 21), fixed with Periodate–Lysine–Paraformaldehyde (PLP) reagent, washed with 5% glycerol, 10% glycerol, and 15% glycerol, and decalcified with EDTA-G for 3 weeks for further tumor tibial lesion histological assessment, as detailed in our previous study [30]. The decalcified tibias were washed with PBS, dehydrated, fixed in paraffin, sectioned, and stained with H&E staining. The tumor lesion area from the stained lung and bone sections was measured with the annotation tool of the ImageScope software (RRID:SCR_014311). For CD8a T cell staining, tumors were harvested from mice (*n* = 4/group) and IHC was carried out using an automated Ventana Discovery Ultra Instrument (Roche, Basel, Switzerland). The slides were deparaffinized and rehydrated. For antigen retrieval, the slides were treated with EDTA buffer and then incubated with primary mouse anti-CD8a antibody (cell signaling, cat# #98941S) at a dilution of 1:70 for 24 min. This was followed by incubation with secondary anti-rabbit antibody conjugated to horseradish peroxidase (HRP). The signal was detected using a DAB chromogen kit. Slides were scanned with Aperio AT Turbo digital. The brown staining indicated CD8a positive staining. Microscopy images (at 400× magnification) were captured randomly using ImageScope and analyzed using Fiji (RRID:SCR_002285). For each sample, five images were captured. Then, in Fiji, images were input and the color deconvolution tool was utilized to separate H&E (total cell stain) and DAB (Cd8a^+^ stain) sections. Next, the analyze particles tool was used to measure the optimal total area of H&E and DAB staining. To calculate the Cd8a^+^ staining area automatically for each image, a macro was created, and images were input one-by-one. Cd8a^+^ staining of T cells percentage was calculated relative to the total cells as [total area of ((DAB/H&E) staining) × 100%)].

### 4.8. Statistical Analysis

Statistical significance was analyzed by two-tailed Student’s T-test and one-way/two-way ANOVA using GraphPad Prism 8 (RRID:SCR_002798).

## 5. Conclusions

Overall, our results showed that while both SAM and anti-PD-1 antibody are effective, the combination of SAM and anti-PD-1 antibody has the greatest effect in reducing tumor growth, progression, and metastasis of a luminal B BCa syngeneic mouse model. Since CPI is already approved for several cancers, including metastatic BCa, and SAM has been extensively shown to block tumor growth and metastasis in several models, including BCa, we propose the potential use of SAM+CPI in patients with luminal BCa. Collectively, results from these studies provide a possible therapeutic strategy combining SAM and CPI (anti-PD-1 antibody) to reduce cancer-associated morbidity and mortality.

## Data Availability

The data analyzed or generated is available within the main file and in the Supplementary Materials.

## Supplementary Materials

The following supporting information can be downloaded at: https://www.mdpi.com/article/10.3390/cancers15010048/s1, Figure S1: Top 10 significantly downregulated pathways obtained from downregulated genes in SAM and anti-PD-1 antibody combination-treated Eo771 tumors versus control tumors. Figure S2: Top 10 significantly upregulated pathways related to immunity obtained from upregulated genes in SAM and anti-PD-1 antibody combination-treated Eo771 tumors versus control tumors. Table S1: List of primers utilized in the study. File S1: List of DEGs (FDR ≤ 0.2) in SAM+anti-PD-1 antibody combination-treated Eo771 tumors versus control.
