# Supplementary material for: Co-Targeting Luminal B Breast Cancer with S-Adenosylmethionine and Immune Checkpoint Inhibitor Reduces Primary Tumor Growth and Progression, and Metastasis to Lungs and Bone
Source: Cancers (Basel). 2022 Dec 22;15(1):48. doi: 10.3390/cancers15010048 (PMC9818024; doi:10.3390/cancers15010048)
Supplement: Supplementary file 1 [file cancers-15-00048-s001.zip › cancers-1982823-supplementary.pdf]

Supplementary Figures

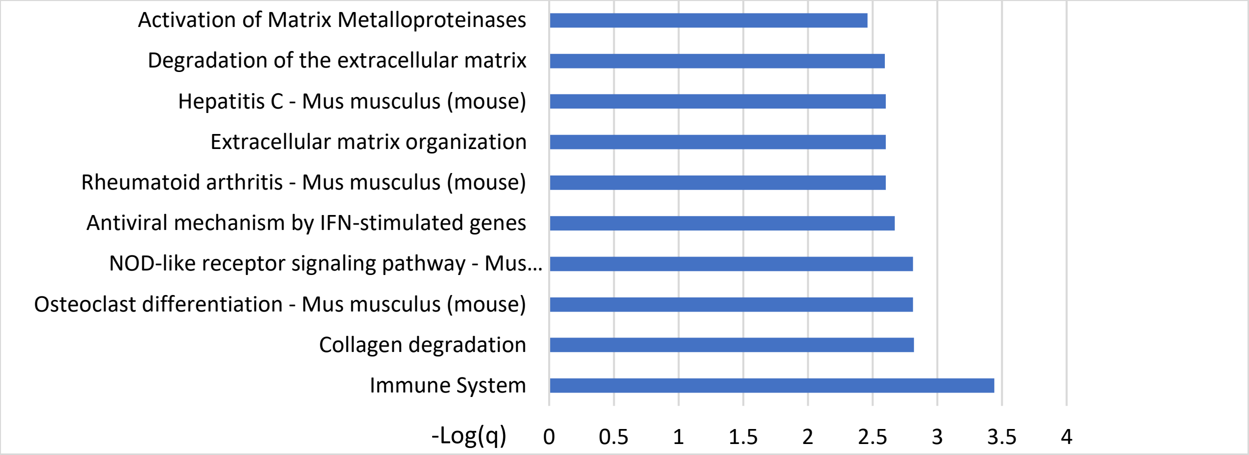

Supplementary Figure S1: Top 10 significantly downregulated pathways obtained from downregulated genes in SAM and anti-PD-1 antibody combination treated Eo771 tumors versus control tumors.

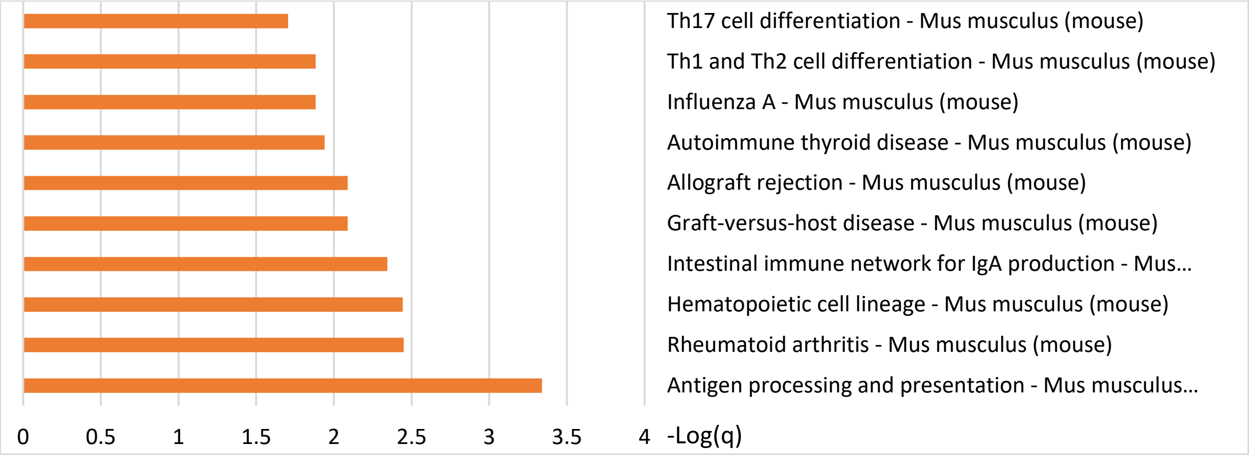

Supplementary Figure S2: Top 10 significantly upregulated pathways related to immunity obtained from upregulated genes in SAM and anti-PD-1 antibody combination treated Eo771 tumors versus control tumors.
